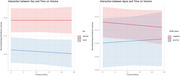# Predicting longitudinal basal forebrain volume in mild cognitive impairment: the role of sex and APOE4 genotype

**DOI:** 10.1002/alz70856_100257

**Published:** 2025-12-24

**Authors:** Alice Grazia, Fedor Levin, Frank Jessen, Michael Wagner, Oliver Peters, Josef Priller, Anja Schneider, Jens Wiltfang, Emrah Düzel, Katharina Buerger, Robert Perneczky, Christoph Laske, Annika Spottke, Alfredo Ramirez, Stefan Teipel

**Affiliations:** ^1^ University Medicine Rostock, Rostock, Mecklenburg‐Vorpommern, Germany; ^2^ German Center for Neurodegenerative Diseases (DZNE) ‐ Rostock/Greifswald, Rostock, Germany; ^3^ Deutsches Zentrum für Neurodegenerative Erkrankungen e. V. (DZNE) Bonn, Bonn, Germany; ^4^ German Center for Neurodegenerative Diseases (DZNE), Bonn, Germany; ^5^ Deutsches Zentrum für Neurodegenerative Erkrankungen e. V. (DZNE) Berlin, Berlin, Germany; ^6^ Department of Psychiatry and Psychotherapy, Charité‐Universitaetsmedizin Berlin, Berlin, Germany; ^7^ German Center for Neurodegenerative Diseases (DZNE), Goettingen, Germany; ^8^ German Center for Neurodegenerative Diseases (DZNE), Magdeburg, Germany; ^9^ German Center for Neurodegenerative Diseases (DZNE), Munich, Germany; ^10^ German Center for Neurodegenerative Diseases (DZNE), Tübingen, Germany; ^11^ Department of Psychosomatic Medicine, Rostock University Medical Center, Rostock, Germany; ^12^ German Center for Neurodegenerative Diseases (DZNE), Rostock, Germany

## Abstract

**Background:**

Imaging studies showed early atrophy of the cholinergic basal forebrain already at prodromal stages of sporadic Alzheimer's disease. It is well known that women and carriers of the APOE4 allele are more likely to develop the disease, however, the mechanisms underlying the role of APOE4 in the pathogenesis of the disease as a whole and at the sex‐specific level are still unknown. In this study we aim at exploring the impact of sex and APOE genotype on longitudinal measures of basal forebrain volume in mild cognitive impairment (MCI) compared to cognitively normal (CN) individuals.

**Method:**

We analyzed MRI scans of individuals from the DELCODE study (mean age: 71 years), comprising 936 CN and 536 MCI at baseline, and 490 CN and 306 MCI at follow‐up (average time:10.88 months). We performed longitudinal volume segmentation and conducted a linear mixed‐effect model to calculate the effect of APOE genotype, sex, diagnosis, time and their interactions over normalized basal forebrain volume.

**Result:**

Sex was a significant predictor of basal forebrain volume (β = ‐0.016, t = ‐3.32, *p* =  0.001)), with male sex and MCI diagnosis (β = ‐0.018, t = ‐6.75, *p* < 0.0001) being significantly associated with lower volume. However, neither APOE status (β = ‐0.008, t = ‐1.39, *p* =  0.165) nor time (β = ‐0.00002, t = ‐0.18, *p* =  0.858) had significant effects, nor did the interactions between sex, APOE status and time (Figure).

**Conclusion:**

Results showed that basal forebrain volume was significantly smaller in males and individuals with MCI, but the rate of change over time did not appear to differ significantly between these groups, ApoE status, or based on sex.